# Emergence and characterization of historically extinct virulent genotype IV Newcastle disease virus in wild and domestic birds: genetic insights, pathogenicity, and vaccine efficacy

**DOI:** 10.1128/jvi.01646-25

**Published:** 2025-11-13

**Authors:** Weiwen Yan, Xinxin Liu, Shanshan Jiang, Hongjin Li, Weiwei Chi, Rui Luo, JiaHuiZi Peng, Feng Jiang, Hongli Li, Tobias Stoeger, Abdul Wajid, Aleksandar Dodovski, Chao Gao, Claro N. Mingala, Dmitry B. Andreychuk, Renfu Yin

**Affiliations:** 1 State Key Laboratory for Diagnosis and Treatment of Severe Zoonotic Infectious Diseases, Key Laboratory of Zoonosis Research, Department of Preventive Veterinary Medicine, Ministry of Education, College of Veterinary Medicine, Jilin University 12510, Changchun, Jilin, China; 2 College of Food Science and Engineering, Jilin University 12510, Changchun, Jilin, China; 3 College of Animal Science, Shanxi Agricultural University 74600, Taiyuan, Shanxi, China; 4 Institute of Lung Health and Immunity (LHI), Comprehensive Pneumology Center (CPC), Helmholtz Zentrum München, Member of the German Center for Lung Research (DZL), Munich, Germany; 5 Department of Biotechnology, Balochistan University of Information Technology, Engineering and Management Sciences 66952, Quetta, Pakistan; 6 Department for Avian Diseases, Faculty of Veterinary Medicine, Ss. Cyril and Methodius University in Skopje 63579, Skopje, Macedonia; 7 Livestock Biotechnology Center, Philippine Carabao Center, Science City of Muñoz 474161 , Muñoz, Philippines; 8 Reference Laboratory for Avian Viral Diseases, FGBI "Federal Centre for Animal Health" (FGBI "ARRIAH"), Vladimir, Russia; University of Kentucky College of Medicine, Lexington, Kentucky, USA

**Keywords:** NDV, genotype IV, LaSota vaccine, migratory birds, phylogenetics

## Abstract

Class II genotype IV Newcastle disease virus (NDV), a historically virulent strain responsible for the first Newcastle disease (ND) panzootic from the 1940s to 1960s, was presumed extinct after its last reported isolate in India in 2000. Here, we report the emergence of four virulent genotype IV NDV isolates from 6,731 wild birds and domestic poultry across nine provinces of China between 2021 and 2023, representing the first genetically confirmed isolation of this ancestral genotype in over two decades. These isolates exhibit remarkably high genetic similarity to ancestral strains, showing minimal divergence despite a temporal span of 50–90 years, yet they differ from the most recently reported isolate from India. Infection with the representative isolate, KS02, caused severe lethality and higher transmissibility in specific-pathogen-free chicks than a genotype VII reference virus. Notably, LaSota vaccination provided only limited protection at the conventional hemagglutination inhibition (HI) threshold and achieved complete protection only when HI titers were at least twofold higher, in contrast to the protection observed against genotype VII challenge. This unusual genetic stability raises concerns about the origin and evolutionary history of these viruses as well as highlights the urgent need to update vaccination strategies, including approaches to elicit higher HI titers through intensified immunization schedules. This study underscores the critical importance of sustained global surveillance of genotype IV NDV in domestic poultry and migratory birds to monitor its spread and evolution.

**IMPORTANCE:**

Virulent Newcastle disease virus (NDV), particularly emerging isolates, poses a major threat to poultry health and production, causing severe morbidity, high mortality, and economic losses. While ancestral class II genotypes II and IX have persisted globally across various bird species, the status of genotype IV NDV—last reported in India in 2000—had been uncertain. This study documents the emergence of genotype IV isolates in wild and domestic birds across China from 2021 to 2023, marking their return after more than two decades of presumed extinction. The representative isolate, KS02, showed severe lethality and high transmissibility in chicks compared to the circulating virus. The LaSota vaccine conferred complete protection against this isolate only when HI titers were at least twofold above the conventional protective threshold. These findings underscore the significant risk posed by reemerging genotype IV NDV, highlighting the urgent need for surveillance and updated vaccination strategies.

## INTRODUCTION

Newcastle disease (ND), caused by virulent strains of Newcastle disease virus (NDV), is a highly contagious viral disease that poses a significant threat to global poultry industries and wild bird populations, with substantial socioeconomic impacts (1, 2). NDV, classified within the genus *Avulavirus* of the family *Paramyxoviridae*, possesses a single-stranded, negative-sense RNA genome of approximately 15.2 kb, encoding six structural proteins: nucleocapsid protein (N), phosphoprotein (P), large RNA-dependent RNA polymerase (L), matrix protein (M), fusion protein (F), and hemagglutinin-neuraminidase (HN) protein (3, 4). These proteins play essential roles in viral replication, pathogenicity, and host immune interactions (5, 6). Based on pathogenicity in chickens, NDV strains are categorized into three pathotypes: lentogenic (avirulent) strains, which are widespread and rarely cause outbreaks; mesogenic strains, which cause respiratory disease and mortality primarily in chickens under 8 weeks; and velogenic (virulent) strains, which cause severe systemic infections with high mortality rates. Due to its high pathogenicity, ND is listed as a notifiable disease in the World Organization for Animal Health (WOAH) *Terrestrial Animal Health Code*, requiring immediate reporting.

NDV exhibits a broad host range and worldwide distribution, classified into two major classes based on the nucleotide sequence of the full-length F gene or complete genome sequences: Class I and Class II. Class I consists of a single genotype, primarily avirulent isolates from wild waterfowl that occasionally spill over into poultry. In contrast, class II displays remarkable genetic diversity, encompassing both virulent and avirulent strains across 22 genotypes (I–XXII) and multiple subgenotypes (7). Historical class II genotypes II, III, IV, and IX, associated with the first ND panzootic from the 1940s to 1960s, are considered “historical” or “ancestral” viruses. Meanwhile, modern virulent Class II NDV genotypes, including V (North America and Africa), VI and VII (worldwide, often linked to recent outbreaks), XI (Madagascar), XII (Asia and South America), XIII (Asia), XIV (Nigeria), XVI (Dominican Republic), and XVII and XVIII (Africa), continue to emerge, underscoring the ongoing evolution of Class II NDV (8–10).

**Fig 1:**
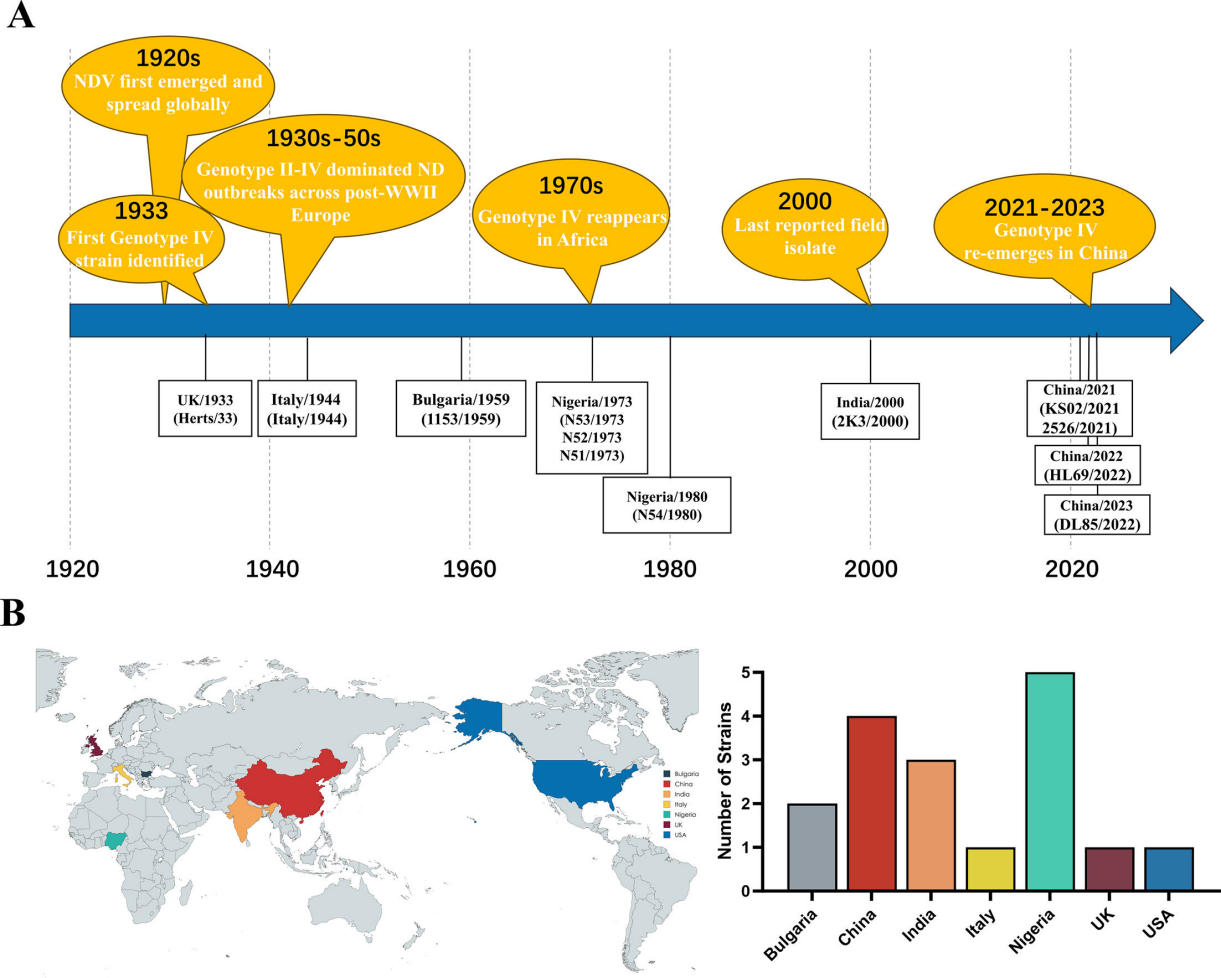
Historical timeline and global distribution of genotype IV NDV. (**A**) Timeline of genotype IV NDV emergence, disappearance, and reemergence. The schematic illustrates the historical trajectory of genotype IV NDV, including its initial discovery, regional spread, disappearance from circulation, and recent reemergence in China. Key historical events are annotated above the timeline, with representative genotype IV strains corresponding to each event listed below. (**B**) Global distribution of all reported genotype IV NDV isolates. This panel summarizes the worldwide occurrence of genotype IV isolates based on published sequence data and isolation records. Each country is labeled with the corresponding number of reported isolates. The map provides an overview of the geographic distribution and temporal detection of genotype IV viruses. Countries and regions are shaded at the national level: the United States, shading includes Alaska. Numeric labels denote the country-level totals of reported genotype IV isolates. Base map: MapChart (mapchart.net); overlays and annotations by the authors.

Class II genotype IV, comprising exclusively virulent strains, was first identified in chickens in Hertfordshire, England, in 1933 (11). From the 1930s to 1950s, genotype IV isolates, including UK/Herts/33, Italian/44, and Bulgaria/Plovdiv/1153/59, dominated Europe during the first ND panzootic. Subsequently, genotype IV spread to Asia, the Middle East, the Americas, and Africa, with notable isolates reported in Nigeria in 1973 and 1980 and the most recent strain, India/NDV-2K3/Pi/2000 (GenBank: FJ986192), identified in India in 2000 (12, 13) (Fig. 1A and B). No genotype IV NDV was reported globally from wild or domestic birds thereafter until this study, which documents the confirmed reemergence of this ancestral lineage in over two decades. This finding represents a significant milestone in tracking the global epidemiology of genotype IV NDV.

The LaSota vaccine, derived from a Class II genotype II NDV strain first isolated in the USA in 1946, is widely used for ND prevention in both commercial and backyard poultry due to its cost-effectiveness, ease of administration, and established efficacy. Notably, HI titers are critical for neutralizing NDV and preventing infection, with levels ≥4 log_2_ generally considered sufficient to protect against virulent NDV (5, 14). However, its protective efficacy against class II genotype IV virulent viruses, particularly the emerging isolates, remains uncertain.

Wild birds, particularly waterfowl, are considered the natural reservoirs for NDV, typically harboring avirulent strains without displaying symptoms. They play a critical role in the global dissemination of NDV, acting as carriers that can transmit the virus to poultry farms during contact, potentially triggering outbreaks. Bidirectional spillover between domestic poultry and wild birds, involving both avirulent and virulent viruses, further complicates NDV dynamics. This study investigates the evolutionary history of recently detected virulent class II genotype IV NDV field isolates in China, evaluates their pathogenicity and transmissibility, and assesses the efficacy of the LaSota vaccine in protecting chicks against these virulent strains.

## RESULTS

### Emergence of genotype IV NDV in migratory birds and domestic poultry along the East Asia–Australasia flyway during 2021 to 2023

**Fig 2:**
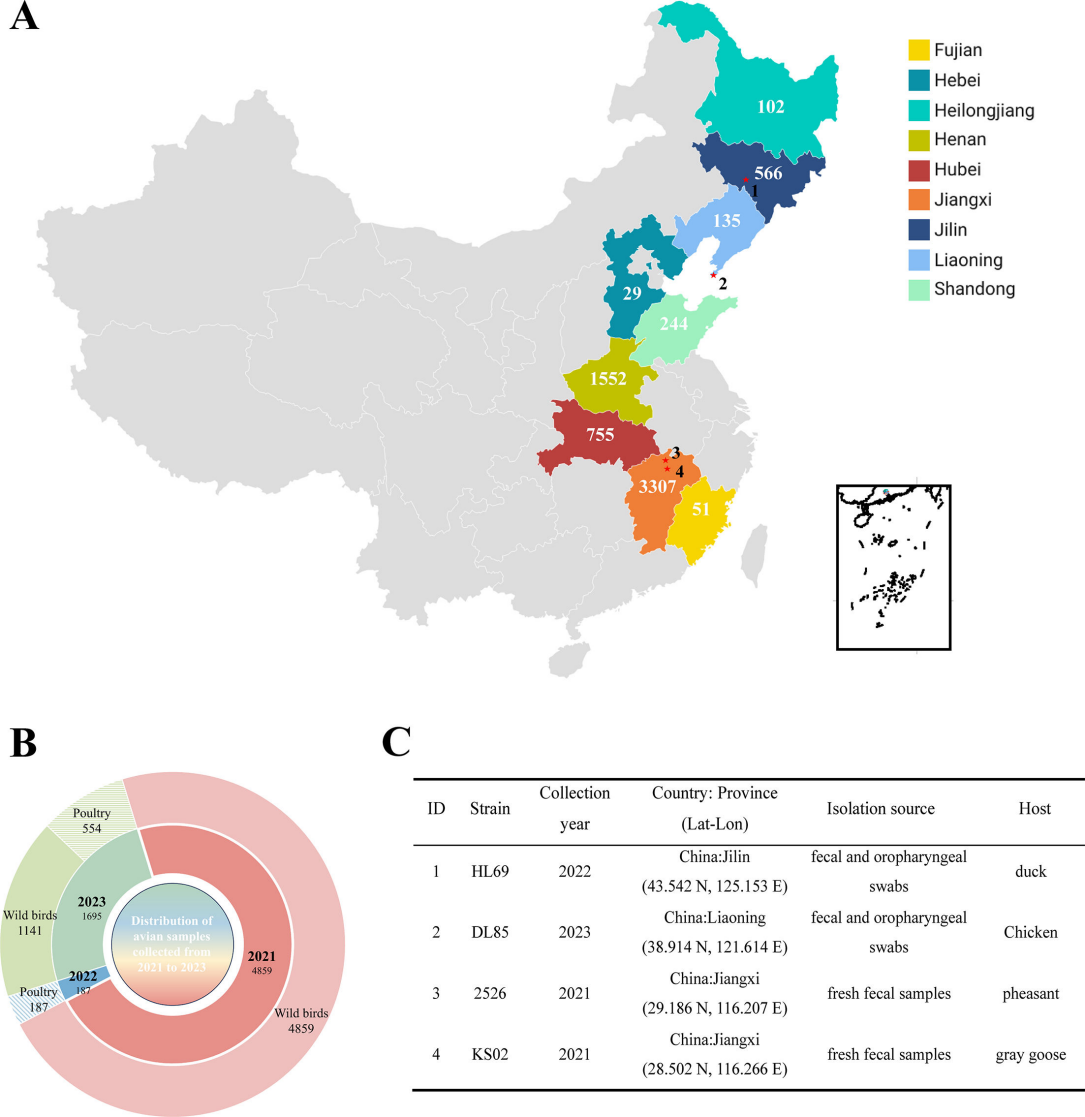
Geographic distribution and classification of avian samples collected in China from 2021 to 2023. A total of 6,731 avian samples were collected from nine provinces across China between 2021 and 2023. (**A**) A total of 6,731 avian samples were collected from nine provinces across China between 2021 and 2023. Provinces are color-coded on the map, with the number of samples indicated within each region. The highest number of samples was collected in Jiangxi (*n* = 3,307), followed by Henan (*n* = 1,552) and Hubei (*n* = 755). Created with Datawrapper; base map/data OpenStreetMap contributors and Natural Earth; overlays by the authors. (**B**) The inset shows a circular diagram summarizing sample classification. The inner ring represents the proportion of samples collected in each year (2021, 2022, and 2023), while the outer ring distinguishes between wild bird and poultry samples. Poultry samples are further marked with hatched lines. Wild bird samples accounted for the majority of total collections, especially in 2021, which alone contributed 72% (4,859/6,741) of all samples. (**C**) Metadata for genotype IV–positive specimens corresponding to starred locations in panel A. The table lists ID, strain, collection year, province with coordinates (Lat–Lon), isolation source, and host (duck, chicken, pheasant, gray goose).

Genotype IV NDV was first identified in England in 1933 and has since been documented across Europe, Asia, the Middle East, the Americas, and parts of Africa (Fig. 1A and B). The most recent report of this ancestral genotype IV virus was from India in 2000 (13), after which its status remained uncertain. Through comprehensive molecular epidemiological surveillance of NDV in 6,731 wild and domestic birds across nine provinces in China from 2021 to 2023 (Fig. 2), this study confirms the reemergence of ancestral genotype IV NDV in both wild and domestic bird populations over two decades.

The first isolate was isolated in 2021 from fresh fecal samples of a wild migratory gray goose (*Anser anser*) at Poyang Lake, Jiangxi Province (longitude 116.266, latitude 28.502), China’s largest freshwater lake and a critical stopover along the East Asia-Australasia Flyway in southeastern China. The second isolate was obtained in 2021 from the fresh feces of a pheasant (*Gallus gallus*) near Poyang Lake (longitude 116.207, latitude 29.186). The third isolate was isolated in 2022 from fecal and oropharyngeal swabs of a domestic duck (*Anas platyrhynchos*) at a live bird market (LBM) in Changchun, Jilin Province (longitude 125.153, latitude 43.542), a city along the same flyway in northeastern China. The fourth isolate was obtained in 2023 from a domestic chicken at an LBM in Dalian, Liaoning Province (longitude 121.614, latitude 38.914), a coastal city on the East Asia-Australasia Flyway in northeastern China. In contrast, no genotype IV NDV was detected in clinical samples from other major wild bird migration routes in China, including the Central Asian Flyway, Southeast Asia-China Flyway, South Asia-China Flyway, and West Pacific Flyway, covering regions such as Hubei, Hunan, Henan, Shandong, Anhui, Qinghai, Inner Mongolia, and Heilongjiang, during the 2021–2023 surveillance period. Additionally, no genotype IV NDV was found in archived clinical samples (pre-2021) stored in our laboratory, despite the presence of other class II genotypes (I, II, VI, and VII) and class I viruses in wild and domestic birds (10, 15, 16). Metadata for the four isolates are presented in Fig. 2.

All genotype IV NDV isolates were confirmed by conventional RT-PCR targeting the F gene of AOAV-1 using established protocols, followed by Sanger sequencing and BLAST similarity search for identification. The isolates were designated as AOAV-1/gray goose/China/KS02/2021 (KS02), AOAV-1/pheasant/China/2526/2021 (2526), AOAV-1/duck/China/HL69/2022 (HL69), and AOAV-1/Chicken/China/DL85/2023 (DL85). Avian influenza virus and other avian avulaviruses were ruled out using the hemagglutination inhibition (HI) assay and RT-PCR, with primer sequences available upon request from the corresponding author. All isolates were successfully propagated in 9- to 10-day-old specific-pathogen-free (SPF) embryonated chicken eggs, and the harvested infectious allantoic fluid tested positive by HA assay, with titers ranging from 256 to 1024 reciprocal dilutions.

To our knowledge, this study represents the genetically confirmed reemergence of ancestral class II genotype IV NDV in wild birds and domestic poultry along the East Asia-Australasia Flyway in China, with no detections in other major migratory routes within the country. This significant finding, following a more than two-decade absence since its last report in India in 2000, highlights the reemergence and persistence of this ancestral NDV genotype, previously presumed extinct, in a critical migratory corridor. These findings provide critical insights into the epidemiological dynamics and potential transmission pathways of genotype IV NDV, emphasizing its importance for regional and global avian disease surveillance.

### Current genotype IV NDV isolates exhibit unexpectedly high genetic similarity to the presumed extinct ancestral strains

**Fig 3:**
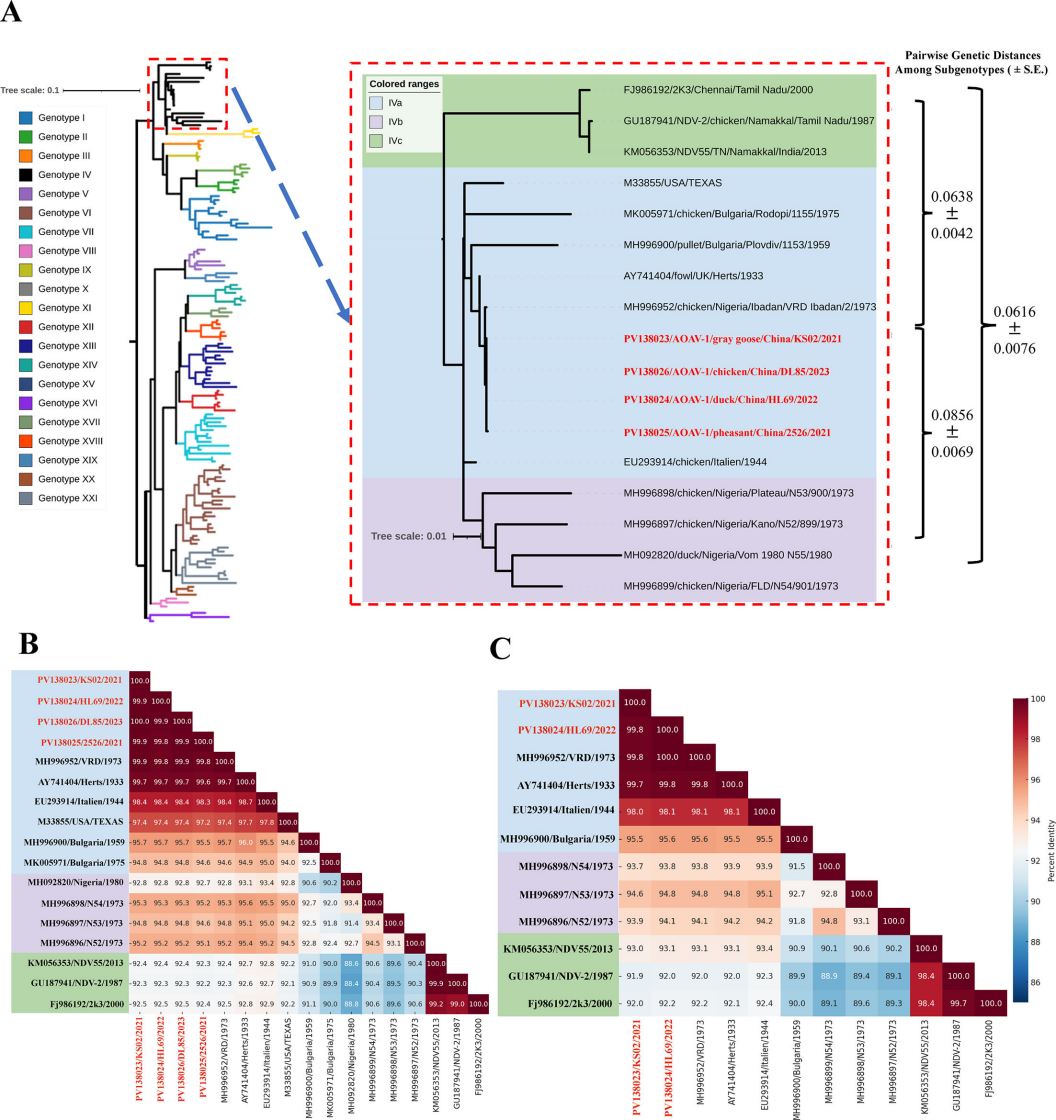
Phylogenetic relationships and genetic similarity of the current genotype IV isolates to historical strains. (**A**) Phylogenetic analysis of NDV genotypes and placement of genotype IV isolates. A maximum likelihood phylogenetic tree was constructed using 126 full-length fusion (F) gene sequences (alignment length: 1,663 nucleotides) of class II NDV strains, encompassing all 21 recognized genotypes (I–XXI). In the accompanying legend, genotypes associated with virulent NDV strains are labeled in red font. The genotype IV clade is highlighted with a red dashed box. An enlarged view of this clade is shown on the right, where subgenotypes IVa, IVb, and IVc are differentiated by green, blue, and purple background shading, respectively. The NDV isolates identified in this study are marked in red. Phylogenetic analysis was conducted using MEGA11, and genotype classification followed established criteria. To the right of the expanded clade, bracketed values indicate the pairwise genetic distances (mean ± s.e.) among subgenotypes IVa, IVb, and IVc. Heatmap of pairwise nucleotide identity (%) based on the full-length F gene (**B**) and hemagglutinin-neuraminidase gene (**C**) sequences among representative NDV strains, including newly isolated genotype IV strains from this study and selected reference strains from other genotypes. In both panels, the color gradient indicates sequence identity percentages, with warmer colors denoting higher similarity. The genotype IVa, IVb, and IVc strains are outlined in blue, purple, and green dashed boxes, respectively. The newly isolated genotype IV strains from China exhibit > 99% identity to genotype IV reference strains from the mid-20th century, highlighting their close genetic relationship and the limited sequence divergence over time.

Phylogenetic analyses of the full-length fusion (F) gene nucleotide sequences of NDV clearly distinguish historical class II genotypes I, II, III, and IV—responsible for the first NDV panzootic in the late 1920s and persisted through the 1950s—from currently circulating genotypes V, VI, VII, and XII to XXI (17, 18) (Fig. 3A). Within the IV genotype, three subgenotypes (IVa, IVb, and IVc) were defined based on full-length F gene sequences, with inter-subgenotype distances ranging from 0.0616 to 0.0856, exceeding the 0.05 divergence threshold (Fig. 3A) (19). All four NDV isolates in this study clustered within subgenotype IVa, showing close genetic similarity to virulent NDV strains isolated in Europe (1930s–1970s), Western Africa (1970s), and the Americas (pre-1980s). Notably, no subgenotype IVb or IVc NDV strains were detected, consistent with their exclusive reports from Nigeria and India, respectively (13, 20). These findings, supported by limited literature on genotype IV NDV, suggest that subgenotype IVa NDV has potential for intercontinental transmission via wild birds, unlike IVb and IVc, which do not exhibit this behavior.

**Fig 4:**
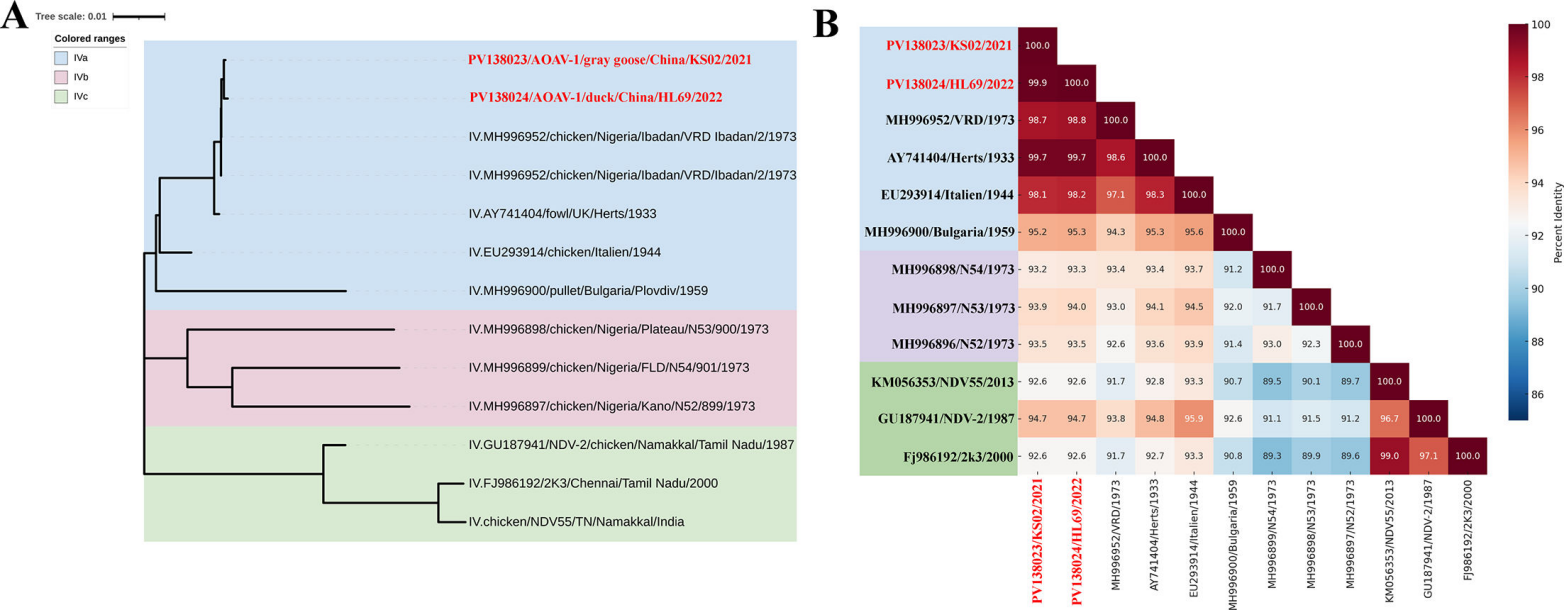
Whole-genome phylogeny and genomic identity of the current genotype IV NDV strains. (**A**) Maximum likelihood phylogenetic tree based on whole-genome sequences of genotype IV NDV strains. Sub-genotypes IVa, IVb, and IVc are highlighted with blue, purple, and green shaded backgrounds, respectively. Two Chinese isolates from this study (KS02 and HL69) are marked in red. Phylogenetic analysis was performed using MEGA11 with the GTR + G + I substitution model and 1,000 bootstrap replicates. (**B**) Heatmap of pairwise whole-genome nucleotide identity (%) among the genotype IV strains. The color gradient reflects sequence similarity, with genotype IVa, IVb, and IVc clades outlined by dashed boxes in matching colors. The Chinese isolates cluster within the IVa subgroup and share nearly complete genomic identity with older IVa strains, underscoring their strong genetic conservation and evolutionary continuity with ancestral genotype IV viruses.

The genotype IV NDV isolates in this study exhibited remarkable nucleotide identity in the F and HN genes, exceeding 94.6% and 95.5%, respectively, with multiple historical subgenotype IVa strains (Fig. 3B and C). For example, divergence from the chicken/VRD Ibadan/1973 was less than 0.2% for both genes, and from the fowl/Herts/1933 strain, it ranged from 0.2% to 0.4%, despite a 50- to 90-year temporal gap. The average evolutionary distance of F gene sequences between current isolates and historical subgenotype IVa strains was 0.0249, significantly lower than distances to IVb (0.0580) and IVc (0.8160) subgenotypes. Bayesian analyses of F gene sequences from 13 genotype IV viruses estimated a mean substitution rate of 6.185 × 10⁻⁵ (standard error: 8.505 × 10⁻⁶). Whole-genome sequencing of two representative isolates, KS02 and HL69, corroborated these findings, confirming a close phylogenetic relationship with historical virulent subgenotype IVa viruses (Fig. 4A). The minimal nucleotide divergence (0.3%–5.7%) across whole-genome sequences within the subgenotype IVa (Fig. 4B), combined with the low F gene substitution rate, suggests an unnatural origin. Despite NDV’s expected evolution rate as an RNA virus, the unusually close genetic similarity between recent subgenotype IVa isolates and ancestral viruses from before the 1970s raises questions about their natural transmission dynamics in domestic poultry or wild bird populations.

### Current genotype IV NDV isolates exhibit high lethality and transmissibility in chicks

All current genotype IV NDV isolates exhibited a characteristic virulent multibasic amino acid sequence (112RRQRRF117) at the fusion (F0) protein cleavage site, consistent with other genotype IV viruses. Their intracerebral pathogenicity index (ICPI) scores exceeded 1.4, and the mean death time (MDT) in eggs was less than 60 hours, further confirming their classification as typical virulent viruses. Among the isolates, KS02 was chosen for further studies due to its status as the first isolate obtained and its high genetic similarity to the other three isolates (Fig. 3B and C and Fig. 4B). In parallel, the genotype VII NDV strain NA-1, a virulent reference strain and the predominant genotype currently circulating in several countries, including China, was used as a control.

**Fig 5:**
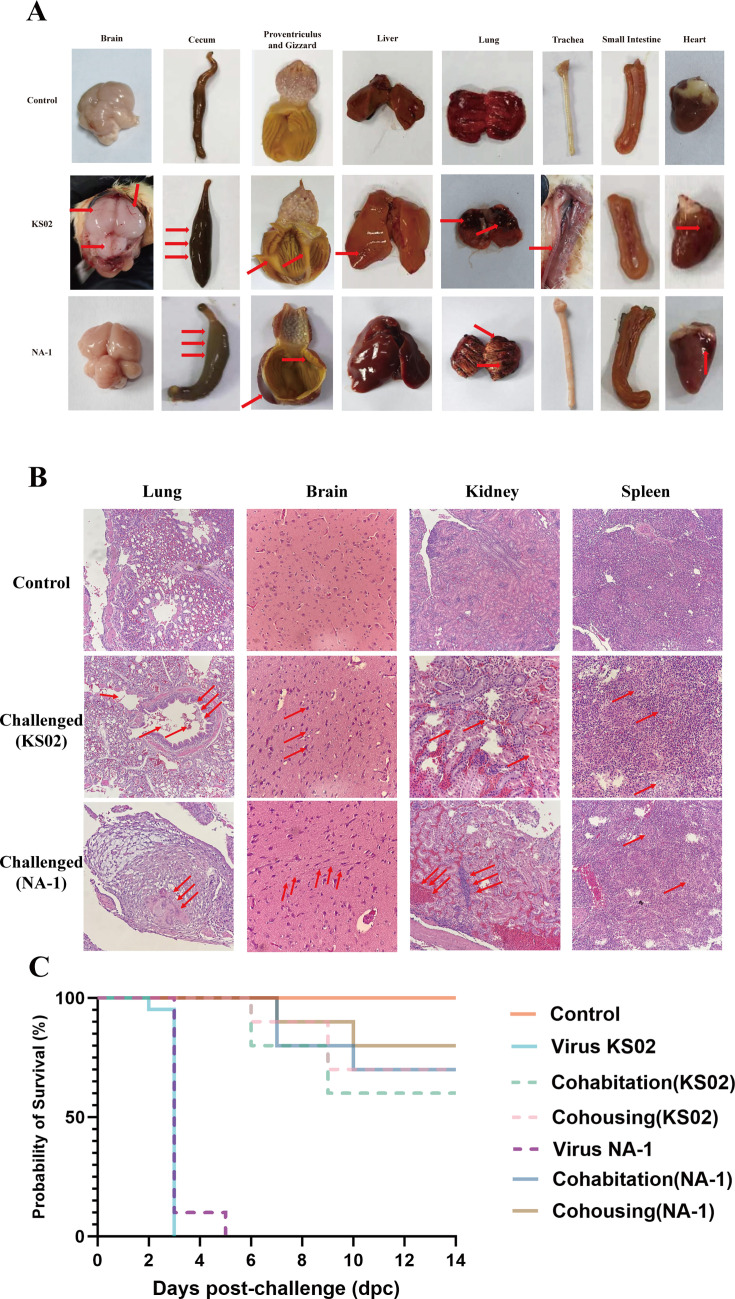
Pathogenicity and transmissibility of the current genotype IV and genotype VII reference NDV in chicks. (**A**) Side-by-side presentation of eight organs across three groups (uninfected control, KS02-challenged, NA-1-challenged) allows direct comparison. Representative gross pathological changes in KS02-challenged chicks include hemorrhagic tracheitis, gelatinous subcutaneous edema in the neck, hemorrhages in the proventriculus and gizzard, renal congestion, pulmonary hemorrhage, hepatic necrosis, splenic infarcts, and cerebral hemorrhage. Red arrows indicate representative lesions in the challenged groups. (**B**) Histopathology (H&E) of lung, brain, kidney, and spleen for the same three groups (rows: Control, KS02, NA-1). Red arrows indicate hallmark changes. (**C**) Survival analysis and transmission assessment. For each virus (KS02, NA-1), four groups were monitored: directly challenged, direct-contact (cohabitation), indirect-contact (cohousing), and the shared uninfected control. All directly challenged chicks succumbed within 3–5 dpc, whereas no mortality occurred in uninfected controls during the 14-day observation. Mortality also occurred in both contact groups, with KS02 showing an earlier onset and higher overall lethality than NA-1.

Twenty SPF chicks were challenged with a single dose of 10⁵ EID_50_ of the KS02 virus via intraocular-nasal drops, resulting in 100% mortality within 2–3 days post-challenge (dpc) (Fig. 5C). Clinical signs of ND infection, including depression, drooping wings, diarrhea, and nasal discharge, appeared approximately 36 hours post-challenge (hpc). Necropsy revealed extensive multi-organ hemorrhages, particularly in the respiratory system, gastrointestinal tract, brain, liver, and heart, along with viscous fluid in the trachea and cloaca in challenged chicks (Fig. 5A). Histopathological analysis identified severe congestion, hemorrhage, and edema in alveolar spaces; infiltration of inflammatory cells (neutrophils and macrophages) in the mucosal layer; non-suppurative encephalitis with neuronal degeneration, perivascular cuffing, and gliosis; lymphoid depletion and congestion in the spleen’s white and red pulp; and tubular necrosis, vacuolar degeneration, and glomerular hypercellularity in the kidney of challenged chicks (Fig. 5B). By comparison, chicks infected with NA-1 died between 2 and 5 dpc and developed clinical signs of ND at approximately 42 hpc—about 6 h later than those infected with KS02—but also exhibited extensive multi-organ hemorrhages (Fig. 5A through C). These findings confirm the systemic spread of the virulent KS02 virus, comparable to the currently predominant genotype VII strains circulating globally, resulting in rapid lethality and severe multi-organ damage.

To evaluate the transmissibility of the KS02 isolate, two SPF chicks were challenged intraocularly and nasally with 10⁵ EID₅₀ of the virus. Twenty naïve SPF day-old chicks were then randomly assigned to two exposure groups: cohabitation (direct contact in the same cage) and cohousing (indirect contact in the same room), with 10 chicks in each group. Clinical signs, oropharyngeal and cloacal swab viral RNA positivity, and survival rates were monitored daily for 15 days post-challenge (dpc). The two challenged chicks died on 3 dpc, serving as the source of infection for both contact groups. Cohabited and cohoused chicks began to exhibit clinical symptoms around 36 hours after exposure, with mortality occurring between 6 and 9 dpc in both groups. Although the cohabited group showed a higher overall mortality rate (40%) compared to the cohoused group (30%), statistical analysis indicated no significant difference between the two groups (Fig. 5C). Viral RNA detection in oropharyngeal and cloacal swabs reached 100% in the cohabited group between 4 and 5 dpc, then declined to 66.7% by 8 dpc and remained stable thereafter. In contrast, the cohoused group showed a lower peak RNA positivity rate of 40% during the same period, which consistently remained below that of the cohabited group throughout the observation period (Table 1). Although naïve chicks cohoused or cohabited with NA-1-infected birds displayed comparable mortality and viral shedding, the onset of mortality and viral RNA detection in swabs was delayed by more than 24 h compared to KS02 (Fig. 5C, Table 2). These findings demonstrate that genotype IV NDV (KS02) exhibits higher virulence than the virulent genotype VII NA-1 strain and is highly transmissible, causing lethal infection not only in directly inoculated birds but also in those exposed through both direct and indirect contact.

**TABLE 1:** Virus shedding in chicks cohabitated or cohoused with KS02-challenged chicks

Days post-challenge	Cohabitation		Cohousing	
	Oropharyngeal (positive/total)	Cloacal (positive/total)	Oropharyngeal (positive/total)	Cloacal (positive/total)
2	0/10	0/10	0/10	0/10
3	7/10	6/10	3/10	2/10
4	10/10	10/10	4/10	4/10
5	8/8	8/8	4/10	4/10
6	6/8	6/8	3/9	3/9
8	4/6	4/6	2/7	2/7
12	4/6	4/6	1/7	1/7
15	4/6	4/6	1/7	1/7

**TABLE 2:** Virus shedding in chicks cohabitated and cohoused with NA-1-challenged chicks

Days post-challenge	Cohabitation		Cohousing	
	Oropharyngeal (positive/total)	Cloacal (positive/total)	Oropharyngeal (positive/total)	Cloacal (positive/total)
2	0/10	0/10	0/10	0/10
3	6/10	5/10	2/10	2/10
4	10/10	9/10	3/10	3/10
5	7/10	6/8	3/10	3/10
6	6/8	5/8	3/9	2/9
8	3/8	4/8	2/9	2/9
12	3/7	4/7	2/8	1/8
15	3/7	3/7	2/8	1/8

### Limited protection of LaSota vaccine-induced HI titers at the minimal protective threshold against the current genotype IV NDV isolate in chicks

The LaSota vaccine, widely used in poultry for its safety, efficacy, and ease of administration via drinking water, injection, or spraying, has historically provided robust protection against ND clinical signs, including respiratory and digestive symptoms, while reducing mortality (5). HI titers are critical for neutralizing NDV and preventing infection, with levels ≥4 log_2_ generally considered sufficient to protect against virulent NDV (5, 14). However, the efficacy of the LaSota vaccine**-**induced HI titers at this minimal protective threshold against the current genotype IV NDV isolates remains uncertain. To assess this, 30 SPF day-old chicks were vaccinated with LaSota vaccine via intraocular-nasal drops, following the manufacturer’s guidelines. Once HI titers against LaSota reached a protective level of at least 4 log₂ in all vaccinated chicks, they were randomly divided into two groups: 20 chicks were challenged with 10⁵ EID₅₀ of the KS02 isolate via intraocular-nasal drops, while 10 chicks received an equivalent dose of phosphate-buffered saline (PBS) as a nonchallenged control. Parallel groups included vaccinated and unvaccinated chicks challenged with the virulent genotype VII NDV strain NA-1 (circulating virus control) as well as unvaccinated, nonchallenged negative controls.

**Fig 6:**
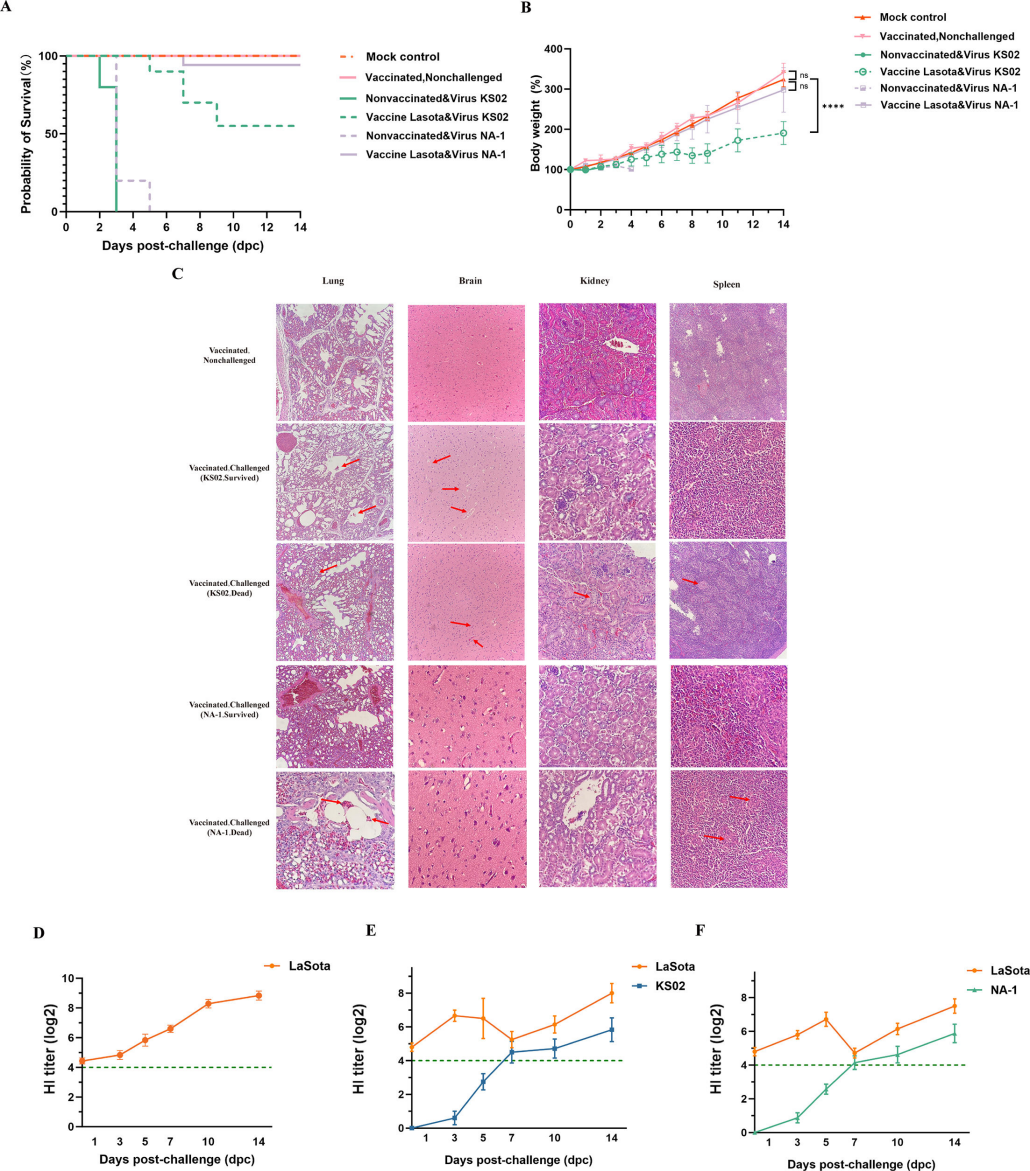
Protective efficacy at the conventional HI protective threshold against KS02 or NA-1 isolates in chicks. (**A**) Kaplan–Meier survival curves of SPF chicks. Vaccinated chicks challenged with the genotype IV strain KS02 showed partial protection (55% survival), while those challenged with the genotype VII strain NA-1 exhibited stronger protection (95% survival). All unvaccinated chicks challenged with KS02 or NA-1 succumbed within 2–5 dpc. All other controls survived the 14-day observation period. (**B**) Body-weight dynamics. Longitudinal body weight expressed as a percentage of baseline (day 0). Vaccinated chicks challenged with KS02 or NA-1 showed growth impairment, more pronounced in the KS02 group. Other controls maintained normal growth. (**C**) Histopathology. Representative H&E-stained sections of lung, brain, kidney, and spleen. Vaccinated survivors after KS02 or NA-1 challenge showed only mild lesions (red arrows), whereas vaccinated birds that succumbed exhibited severe pathological changes, including pulmonary hemorrhage, neuronal necrosis, nephritis with tubular damage, and splenic hemorrhage with lymphoid depletion. (**D**) LaSota-specific HI titers in vaccinated, nonchallenged chicks. HI titers remained above the 4 log₂ threshold during the 14-day observation period. (**E**) HI titers in vaccinated chicks challenged with KS02. LaSota-specific HI titers remained above 4 log₂ and rose further post-challenge, while KS02-specific HI titers were initially undetectable and increased progressively. (**F**) HI titers in vaccinated chicks challenged with NA-1. LaSota-specific HI titers remained consistently high and showed a modest anamnestic rise after challenge. NA-1-specific HI titers were initially undetectable, began increasing from 3 dpc, and rose progressively by 14 dpc, although overall levels remained lower than LaSota-specific titers. Data are presented as mean ± SD; the dashed line indicates the 4-log₂ threshold.

Consistent with KS02 pathogenicity results (Fig. 5C), all unvaccinated chicks challenged with either KS02 or NA-1 died within 5 dpc, whereas the nonchallenged negative and vaccinated-nonchallenged controls remained healthy throughout the observation period (Fig. 6A). Among vaccinated chicks challenged with NA-1, no obvious clinical signs were observed, and only 1 of 20 (5 %) died at 7 dpc. In contrast, vaccination conferred only partial protection against KS02: although no clinical signs were observed within the first 3 dpc, 9 of 20 vaccinated chicks died between 5 and 9 dpc (2, 4, and 3 deaths on 5, 7, and 9 dpc, respectively), resulting in a 55% survival rate (Fig. 6A). Surviving vaccinated chicks challenged with KS02 exhibited significantly slower growth rates from 4 dpc onward (Fig. 6B) and displayed persistent ND-like clinical signs, including head shaking, lethargy, reduced appetite, and respiratory distress, throughout the 14-day observation period. Pathological examination of surviving KS02-challenged chicks revealed mild tissue damage, including slight congestion and inflammatory cell infiltration in the lung bronchi, mild neuronal degeneration in the brain (characterized by cell shrinkage and occasional loss without significant structure disruption), slight vacuolization in kidney tubular epithelial cells, and moderate lymphocyte depletion in the spleen’s white pulp (Fig. 6C). These changes were less severe than those observed in naïve challenged chicks and non-surviving vaccinated and KS02-challenged chicks (Fig. 6C and 5B). No significant lesions were detected in the vaccinated-nonchallenged, vaccinated-NA-1-challenged, and negative control groups over the 14-day period.

Despite 55% survival after a single LaSota dose, oropharyngeal virus shedding was detected in 60% of vaccinated chicks challenged with KS02 (12/20) at 3 dpc, gradually declining and becoming undetectable by 14 dpi (0/11). Cloacal shedding was observed in 15% of chicks (3/20) at 3 dpc and was not detected thereafter (Table 3). In contrast, vaccinated chicks challenged with NA-1 showed significantly lower shedding rates and shorter duration (Table 3).

**TABLE 3:** Viral shedding in chicks with the LaSota vaccine-induced HI titers at this minimal protective threshold (≥4 log₂) following challenge with KS02 or NA-1

Days post-challenge	KS02		NA-1	
	Oropharyngeal (positive/total)	Cloacal (positive/total)	Oropharyngeal (positive/total)	Cloacal (positive/total)
1	20/20	8/20	6/20	3/20
3	12/20	3/20	4/10	3/20
5	4/18	0/18	2/10	0/20
7	4/14	0/14	0/19	0/19
9	1/11	0/11	0/19	0/19
11	1/11	0/11	0/19	0/19
14	0/11	0/11	0/19	0/19

Because HI titers are pivotal for NDV neutralization, sera from vaccinated nonchallenged and vaccinated challenged chicks were tested with LaSota and KS02 viruses at 1, 3, 5, 7, 10, and 14 dpc. Vaccinated nonchallenged chicks showed robust LaSota-specific HI titers but minimal or undetectable KS02-specific titers (Fig. 6D). In vaccinated challenged chicks, LaSota-specific HI titers initially declined from a peak between 3 and 7 dpc, then sharply increased, reaching a second peak by 14 dpc, while KS02- and NA-1-specific HI titers rose steadily throughout the observation period (Fig. 6E and F). Despite LaSota-specific HI titers remaining above the minimal protective threshold of 4 log_2_, the LaSota vaccine provided limited protection, with only a 55% survival rate against the current genotype IV KS02, compared with its stronger protection against NA-1 over the 14-day period (Fig. 6A). Collectively, these results indicate that the LaSota vaccine-induced HI titers at minimal protective threshold offer only limited protection against clinical signs, tissue damage, growth retardation, and virus shedding caused by the current genotype IV NDV isolate, in contrast to the stronger protection observed against the genotype VII strain NA-1.

### LaSota vaccine confers complete protection against the current genotype IV NDV isolate when HI titers exceed the protective threshold twofold

Because HI titers are pivotal for NDV neutralization, and LaSota-induced HI titers at the minimal protective threshold provide only limited protection against the current genotype IV NDV isolate, we next examined whether the LaSota vaccine can confer complete protection when the HI titers are twofold (≥5 log₂) or even fourfold (≥6 log₂) above the protective threshold. Once vaccinated chicks achieved HI titers either ≥5 to <6 log_2_ or ≥6 log₂, they were randomly allocated to four groups. In all, 20 chicks (10 from each titer category) were challenged with 10⁵ EID₅₀ of the KS02 isolate via intraocular-nasal inoculation, while 10 chicks (five from each titer category) received an equivalent dose of PBS as nonchallenged controls. Parallel groups included unvaccinated chicks challenged with KS02 and unvaccinated nonchallenged negative controls.

**Fig 7:**
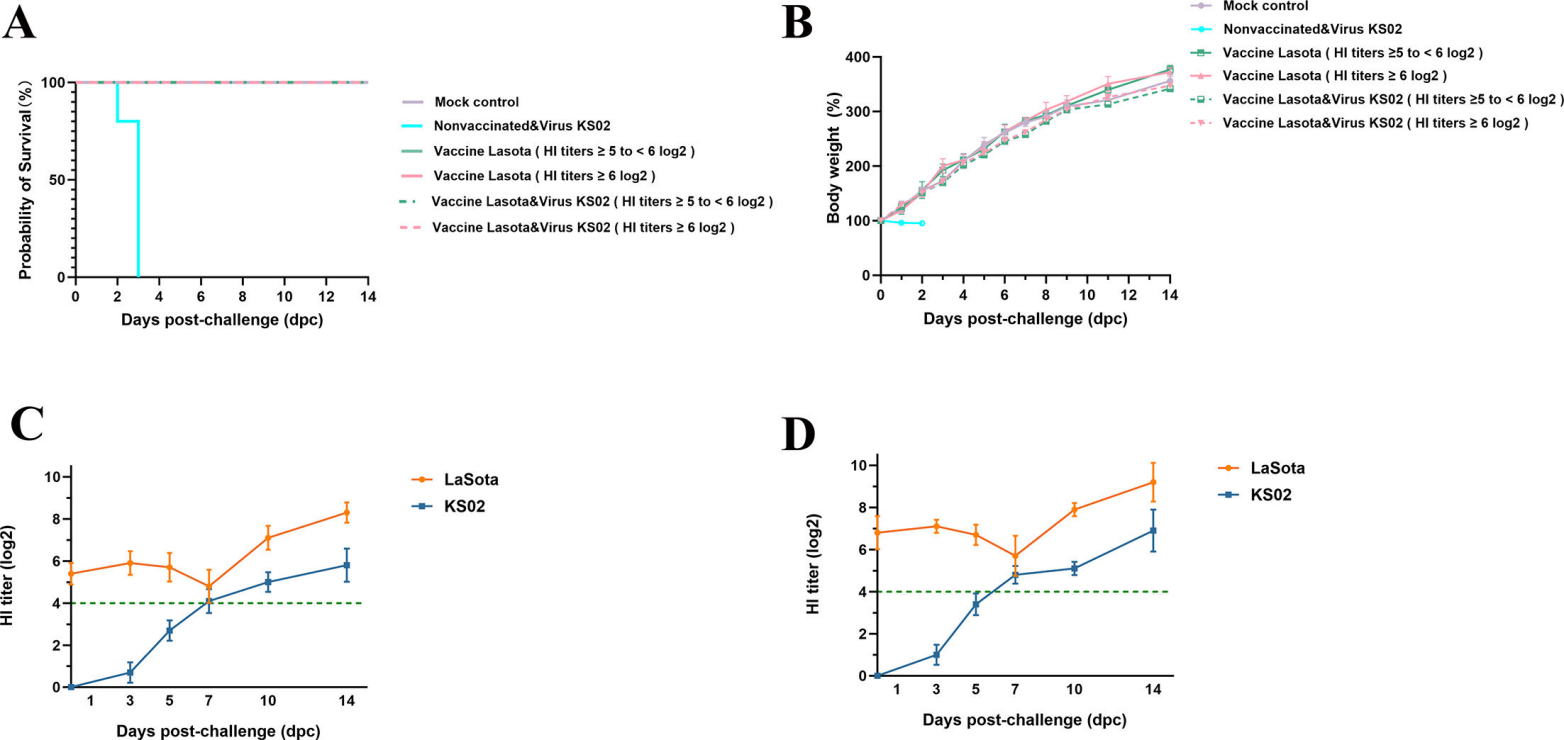
LaSota vaccine confers complete clinical protection against genotype IV NDV (KS02) when HI titers exceed the protective threshold by at least twofold. (**A**) Kaplan–Meier survival curves of SPF chicks. All unvaccinated challenged chicks succumbed within 2–3 dpc, whereas every vaccinated bird with pre-challenge HI titers ≥ 5 to <6 log_2_ or ≥ 6 log₂, as well as vaccinated, nonchallenged, and mock controls, survived the 14-day observation period. (**B**) Body weight dynamics. Vaccinated, nonchallenged, and mock control groups maintained normal growth, and only two vaccinated, challenged chicks showed mild and transient growth impairment compared with controls. (**C**) HI titer kinetics in vaccinated, challenged chicks with pre-challenge HI ≥ 5 to <6 log₂. LaSota-specific HI titers remained above the 4 log₂ threshold and rose further after challenge, while KS02-specific HI titers were initially undetectable but increased progressively from day 3 onwards. (**D**) HI titers kinetics in vaccinated, challenged chicks with HI ≥ 6 log₂. These chicks exhibited patterns similar to panel C but maintained consistently higher LaSota- and KS02-specific HI titers across the observation period. Data are shown as mean ± SD.

In the experimental setup, all unvaccinated, challenged birds began showing clinical signs—including depression, dyspnea, anorexia, and watery greenish-white diarrhea—by 36 hpc and died within 2–3 dpc. In contrast, no obvious clinical signs or mortality were observed in any vaccinated birds with an HI titer of either ≥5 to <6 log_₂_ or ≥6 log_₂_ through 14 dpc observation period, nor in the unvaccinated, nonchallenged controls (Fig. 7A and B). Notably, virus shedding in vaccinated birds was largely restricted to the first 7 days dpc, with the exception of four birds with HI titer ≥5 to <6 log_₂_ and two birds with titer ≥6 log_₂_ (Table 4).

**TABLE 4:** Virus shedding in LaSota-vaccinated chicks with HI titers twofold (≥5 to <6 log₂) or fourfold (≥6 log₂) above the protective threshold (4 log_2_) after KS02 challenge

Days post-challenge	HI titer with ≥5 to <6 log₂		HI titer with ≥6 log₂	
	Oropharyngeal (positive/total)	Cloacal (positive/total)	Oropharyngeal (positive/total)	Cloacal (positive/total)
1	4/10	0/10	2/10	0/10
3	2/10	0/10	1/10	0/10
5	1/10	0/10	0/10	0/10
7	0/10	0/10	0/10	0/10
9	0/10	0/10	0/10	0/10
11	0/10	0/10	0/10	0/10
14	0/10	0/10	0/10	0/10

Moreover, there was no significant difference in either the number of shedders or the duration of shedding between the two KS02-challenged strata (HI ≥6 log₂ vs HI ≥5 to <6 log₂; *P* > 0.05). In contrast, both higher-titer strata shed significantly less virus and cleared earlier than the minimal-threshold vaccinated group challenged with KS02 (HI ≥4 log₂; *P* < 0.05). Collectively, these findings indicate that the LaSota vaccine provides complete protection against the current genotype IV NDV isolate when HI titers exceed the protective threshold by at least twofold.

## DISCUSSION

The confirmed emergence of a died-out historical virulent genotype IV NDV in wild and domestic birds over two decades raises serious concerns for poultry health. This study documents the isolation of four genotype IV NDV isolates from migratory geese, pheasants, ducks, and chickens between 2021 and 2023, marking the emergence of a lineage presumed extinct since its last documentation in India in 2000. Phylogenetic analyses place these isolates within subgenotype IVa, revealing an unexpectedly high genetic similarity to virulent strains from the 1930s to 1940s, with nucleotide divergence as low as 0.3%–2.9% (Fig. 4B). This minimal genetic divergence over a 50- to 90-year span is remarkable for an RNA virus like NDV, which typically evolves rapidly (10^-3^ to 10^-4^ substitutions per site per year) due to the error-prone nature of its RNA-dependent RNA polymerase (21–23). Bayesian analysis estimated a mean substitution rate of 6.185 × 10^-5^ for the F gene, suggesting an unusually slow evolutionary rate. The global distribution of subgenotype IVa, previously reported in Europe, Africa, Asia, and the Americas, underscores its potential for intercontinental spread via migratory birds, unlike subgenotype IVb and IVc, which are restricted to West Africa and India. This remarkable genetic stability of these isolates, coupled with detection gaps exceeding 20 years (last report in India in 2000, Fig. 1A), aligns with several non-mutually exclusive hypotheses, including reintroduction from preserved material, persistence in under-sampled ecological niches, or repeated introductions via migratory routes. As our data are observational, these remain hypotheses. No event-level climatic drivers were identified in our sampling records; rigorous attribution would require dedicated environmental data sets and modeling. Further investigation into ecological and anthropogenic factors, including wild bird populations, is critical to understanding this emergence.

All isolates exhibited a virulent multibasic cleavage site (112RRQRRF117) in the F protein, an ICPI score above 1.4, and an MDT of less than 60 hours, confirming their velogenic nature. The pathogenicity and transmissibility of the representative isolate, KS02, further underscore the threat posed by these genotype IV NDV strains. Experimental infection with KS02 in SPF chicks caused 100% mortality within 2–3 days, with severe multi-organ damage, including hemorrhages in the respiratory and gastrointestinal systems, non-suppurative encephalitis, and lymphoid depletion in the spleen (Fig. 5). These findings align with the systemic spread typical of velogenic NDV strains (24–27). Additionally, KS02 demonstrated high transmissibility, with a 40% mortality rate in cohabitated chicks and 100% viral RNA detection in oropharyngeal and cloacal swabs within 4–5 days post-cohabitation (Fig. 5C and Table 1). The delayed onset of clinical signs and reduced viral shedding in cohoused chicks compared with directly cohabitated ones suggests that direct contact is a more significant transmission route than environmental exposure, consistent with known NDV dynamics (27). In contrast, chicks either directly challenged with the virulent reference strain NA-1 or cohoused/cohabited with NA-1-infected birds displayed similar mortality and morbidity, but both the onset of death and the appearance of viral shedding were delayed compared with KS02 (Fig. 5C, Table 2). Collectively, these findings demonstrate that genotype IV NDV (KS02) is more virulent than the virulent genotype VII NA-1 strain and is highly transmissible, causing lethal infection not only in directly inoculated birds but also in those exposed through both direct and indirect contact. Notably, these results emphasize the need for enhanced biosecurity measures in LBM and farms along migratory flyways, particularly the East Asia-Australasia Flyway, which facilitates viral spread through migratory waterfowl.

The LaSota strain (Class II, Genotype II) is one of the most widely used poultry vaccines, and the vaccine-induced HI titers of ≥4 log_2_ are generally considered sufficient to protect against virulent NDV (5, 14). However, the effectiveness of LaSota against current genotype IV NDV isolates remains uncertain. Despite maintaining HI titers at the conventional protective threshold of 4 log2, the vaccine achieved only a 55% survival rate in KS02-challenged chicks, with survivors exhibiting persistent clinical signs, growth retardation, and mild tissue damage, including lung congestion, neuronal degeneration, and spleen lymphocyte depletion (Fig. 6). All vaccinated and challenged chicks exhibited viral shedding, with oropharyngeal shedding persisting throughout the 14-day observation period. In the KS02-challenged group, cloacal shedding was detected in 40% of chicks at 1 dpc (Table 3). Complete protection against the current genotype IV NDV isolate was achieved only when LaSota-specific HI titers exceeded the protective threshold by at least twofold. Under these conditions, both the rate and duration of viral shedding reduced in an HI titer-dependent manner (Fig. 7C and D, and Table 4). These observations are consistent with previous reports that higher HI titers are required to fully block infection or virus shedding by virulent heterologous NDV strains (14, 28–30). In contrast, vaccinated chicks with minimal protective HI titers of 4 log_2_ that were challenged with the NA-1 strain showed no obvious clinical signs or significant lesions, shed markedly less virus for a shorter duration, and experienced only 1 of 20 deaths (5%) by 7 dpc (Fig. 6A and Table 3). Collectively, these findings highlight the critical role of robust humoral immunity in cross-protection against emergent virulent NDV lineage and underscore the need to maintain HI titers at least twofold above the conventional protective threshold to effectively control outbreaks of potential virulent isolates such as the genotype IV NDV described in this study.

Migratory waterfowl, such as the gray goose (*Anser anser*), likely play a key role in maintaining and disseminating these viruses, as evidenced by the initial isolation from fecal samples at Poyang Lake, a critical stopover site. The absence of genotype IV NDV in other flyways during 2021–2023 indicates that host susceptibility, environmental conditions, or viral fitness may influence its distribution. Whether the genotype IV NDV described in this study occupies a distinct ecological niche remains uncertain and warrants further investigation. Notably, bidirectional spillover between wild birds and domestic poultry, demonstrated by isolates from wild geese as well as domestic ducks and chickens, complicates control efforts. Therefore, a One Health approach, integrating surveillance of wild and domestic bird populations, is essential to monitor and mitigate NDV spread across ecological interfaces. The involvement of migratory birds also raises concerns about the international spread of genotype IV NDV, as these birds can disseminate the virus across continents via established flyways, posing a global risk to poultry industries and food security.

In conclusion, the emergence of genotype IV NDV in wild and domestic birds after over two decades presents a significant challenge to poultry health and production due to its high pathogenicity, efficient transmissibility, and the limited protection provided by LaSota-induced HI titers at the conventional protective threshold. The unusual genetic stability of these isolates raises critical questions about their origin and evolutionary history, necessitating further genomic and epidemiological studies. Notably, the observation that the LaSota vaccine confers complete protection only when HI titers reach at least twice the conventional protective level highlights the urgency of updating vaccination strategies, including efforts to elicit higher HI titers via intensified immunization schedules. Enhanced surveillance along migratory flyways, strengthened biosecurity measures, and a One Health framework are essential to prevent future ND outbreaks and address the broader implications of emerging ancestral strains for global poultry industries. Together, these findings highlight the continuing risk posed by divergent NDV strains and the necessity of reassessing vaccine and surveillance strategies for newly emerged genotypes.

## MATERIALS AND METHODS

### Sample collection

From 2021 to 2023, a total of 6,731 samples, comprising fresh fecal droppings from wild migratory birds at stopover points and oropharyngeal and cloacal swabs from domestic poultry in live bird markets (LBM), were collected across nine provinces in China (Hubei, Hunan, Henan, Hebei, Shandong, Anhui, Jilin, Liaoning, and Heilongjiang) (Fig. 1). Samples were collected under sterile conditions using swabs placed in 2 mL EP tubes containing 1.5 mL of viral transport medium (40% glycerol, 2,000 U/mL penicillin, 2 mg/mL streptomycin, 50 µg/mL gentamycin, 50 U/mL nystatin, and 0.5% bovine serum albumin). During field collection, samples were kept on dry ice and subsequently stored at −80°C upon arrival at the laboratory.

### Virus isolation

Samples were inoculated into the allantoic cavities of 9- to 10-day-old SPF chicken embryos (Jinan SAIS Poultry Company, Shandong, China) in accordance with the WOAH standard manual for ND detection. Allantoic fluids were harvested upon embryo death or at the end of the incubation period and tested for NDV using hemagglutination (HA) assays and RT-PCR to detect NDV-specific nucleic acid signatures. Hemagglutinated allantoic fluids were stored at −80°C for subsequent analysis.

### RNA extraction, RT-PCR, whole-genome sequencing, and phylogenetic analysis

RNA extraction, RT-PCR, and whole-genome sequencing were performed following the protocols and conditions described in our previous studies (15, 31, 32). Briefly, viral RNA was extracted from allantoic fluid using TRIzol Reagent (Sigma, Shanghai, China) according to the manufacturer’s instructions. Reverse transcription and PCR were subsequently conducted using a reverse transcription kit (Novoprotein, Suzhou, China) and a 2× M5 SuperLong Taq MasterMix kit (Mei5Bio, Beijing, China), respectively, to amplify the NDV F gene, adhering to previously established protocols (15, 31, 32). Purified RT-PCR products were sequenced using an ABI 3730XL automated DNA analyzer (Applied Biosystems, Massachusetts, USA). A BLAST similarity search confirmed NDV identity, and NDV-positive samples were subjected to further sequencing to obtain F gene or whole-genome sequences.

Whole-genome sequencing of the KS02 and HL69 isolates was performed as previously described (31, 32), while the full-length F and HN gene sequences of the 2526 and DL85 isolates were obtained through RT-PCR and Sanger sequencing (30). Phylogenetic trees were constructed using MEGA 11 software (33, 34) based on full-length F gene and whole-genome sequences of NDV across various genotypes. Maximum Likelihood (ML) trees were constructed using the Kimura 2-parameter model, incorporating a discrete Gamma distribution (+G) and invariant sites (+I). Bootstrap analysis with 1000 replicates was used to assess node support. All trees were drawn to scale, with branch lengths representing the number of substitutions per site. Analyses included codon positions 1st, 2nd, and 3rd, as well as noncoding regions; gaps and missing data were excluded. The final data set for the F gene alignment comprised 1,662 nucleotide positions.

### Pathogenicity tests and EID50 assays

The virulence of the Chinese genotype IV NDV isolates was determined using the ICPI per WOAH standards for ND. Briefly, 10-day-old SPF chicks were inoculated intracerebrally with 0.05 mL of allantoic fluid serially diluted 10-fold in sterile PBS. Birds were examined once daily for 8 consecutive days and scored 0 (normal), 1 (sick), or 2 (dead); the ICPI was calculated as the mean daily score per bird over the 8-day observation period, with higher values indicating greater virulence. MDT was assessed by inoculating 9- to 10-day-old SPF embryonated chicken eggs with serially diluted allantoic fluid (10-fold in PBS). A volume of 0.1 mL was inoculated into the allantoic cavity of 5–6 embryos per dilution, and MDT was defined as the mean time to death (in hours) among embryos receiving the minimal lethal dose. Eggs were candled twice daily to monitor embryonic death. Viruses causing death within 60 hours were classified as velogenic, 61–90 hours as mesogenic, or beyond 90 hours as lentogenic (35). The 50% embryo infectious dose (EID50) for the KS02 was determined using the Reed-Muench method. EID50 titers were expressed as log_10_ EID50/mL, and back-titrations were performed for all challenge stocks.

### Challenge and transmission studies of genotype IV (KS02) and Genotype VII (NA-1) NDV in SPF chicks

Sixty-five SPF day-old chicks were divided into five groups. Two challenge groups (20 chicks each) were inoculated via ocular and nasal routes with 10⁵ EID₅₀/100 µL of KS02 or NA-1, respectively. To evaluate transmission capacity, two SPF chicks from each challenge group were randomly selected and used as seeders for subsequent exposure experiments. For each virus, two contact groups of uninfected chicks (*n* = 20) were established: one group (*n* = 10) was cohoused in the same room for indirect contact, and the other group (*n* = 10) was placed in the same cage for direct contact with a seeder chick. The remaining five chicks served as an unchallenged negative control group. All chicks were monitored daily over a 15-day period post-challenge. Oropharyngeal and cloacal swabs were collected daily to assess viral shedding by RT-PCR, and clinical signs and survival were recorded.

### Evaluation of LaSota vaccine protective efficacy at the conventional HI protective threshold against KS02 or NA-1 isolates in chicks

For each virus, 30 chicks were vaccinated with a single dose of the LaSota vaccine per manufacturer’s instructions. Once HI titers against LaSota virus reached a minimum of 4 log₂, the chicks were divided into two groups. The challenge group (20 chicks) received 100 µL of 10⁵ EID50 of either KS02 or NA-1 via ocular and nasal routes, while 10 chicks served as vaccinated, unchallenged controls. Chicks were observed for 14 dpc, with daily oropharyngeal and cloacal swabs collected to monitor viral shedding. HI antibody titers against LaSota, NA-1, and KS02 were measured throughout the observation period. An additional group of 10 unvaccinated, unchallenged chicks served as mock controls.

### Evaluation of LaSota-mediated protection against KS02 at elevated HI titers

Although LaSota-induced HI titers at the minimal protective threshold provide only limited protection against the current genotype IV NDV isolate, we next assessed whether higher HI titers could confer complete protection. Specifically, we examined protection when the LaSota-specific HI titer was twofold (≥5 log₂) or fourfold (≥6 log₂) above the conventional threshold. Once vaccinated chicks reached pre-challenge HI titers of either ≥5 to <6 log₂ or ≥6 log₂, they were randomly allocated into four groups. For each titer level, 10 chicks were challenged with 100 µl of 10⁵ EID50 of KS02 via ocular and nasal routes, while 5 chicks served as vaccinated, unchallenged controls. In parallel, 5 unvaccinated chicks challenged with KS02 and 10 unvaccinated, unchallenged chicks served as additional controls. Clinical signs, survival, body weight, HI titers, and virus shedding were monitored as described above.

### Hemagglutination (HA) and HI assays

HA and HI assays were performed using the WOAH-recommended standard microtiter plate method without any modifications. For the HI test, 4 HA units of either the LaSota strain or the KS02 isolate were used to measure HI titers against each virus.

### Histopathological analysis

Tissue samples from chicks that died due to infection or were selected for analysis were fixed, sectioned, and subjected to histopathological examination. Lesions were documented to characterize pathological changes associated with NDV infection.

### Statistical analysis

Statistical analyses were conducted using GraphPad Prism version 10.1.2. Data from three independent experiments are expressed as mean ± SD. Statistical significance was determined using an unpaired two-tailed Student’s t-test, with significance levels denoted as * *P* < 0.05, ** *P* < 0.01, *** *P* < 0.001.

## Data Availability

All data generated or analyzed during this study are included in the published article. The complete genome sequences of the KS02 and HL69 isolates, along with partial genome sequences of the 2526 and DL85 isolates, have been deposited in GenBank under accession numbers PV138023 (KS02), PV138024 (HL69), PV138025 (2526), and PV138026 (DL85).
